# Neurophysiological correlates of object recognition in the dorsal subiculum

**DOI:** 10.3389/fnbeh.2012.00046

**Published:** 2012-07-19

**Authors:** Eric H. Chang, Patricio T. Huerta

**Affiliations:** ^1^Laboratory of Immune and Neural Networks, Center for Biomedical Science, The Feinstein Institute for Medical Research, North Shore-LIJ Health System, ManhassetNY, USA; ^2^Department of Molecular Medicine, Hofstra North Shore-LIJ School of Medicine, ManhassetNY, USA

**Keywords:** novel object, theta, gamma, coherence, place cell, mouse

## Abstract

The medial temporal lobe (MTL) encompasses a network of interconnected cortical areas that is considered the neural substrate for some types of memory, such as spatial, episodic, recognition, and associative memory. Within the MTL, the subiculum has been well characterized in terms of its connectivity and structure, but its functional role remains elusive. A long-held view is that the subiculum is mainly involved in spatial encoding because it exhibits spatially selective firing and receives prominent projections from the CA1 field, which is an essential substrate for spatial memory. However, the dorsal subiculum (DS) is also reciprocally connected to the perirhinal and postrhinal cortices, which are critically involved in recognition memory. This connectivity pattern suggests that DS might encode not only spatial signals but also recognition signals. Here, we examined this hypothesis by recording with multi-electrodes in DS and CA1 of freely behaving mice, as they performed the novel object recognition (NOR) task. Analysis of network oscillations revealed that theta power was significantly higher in DS when mice explored novel objects as compared to familiar objects and that this theta modulation was absent in CA1. We also found significant differences in coherence between DS and CA1, in the theta and gamma bands, depending on whether mice examined objects or engaged in spatial exploration. Furthermore, single-unit recordings revealed that DS cells did not exhibit phase-locked firing to theta and differed from CA1 place cells in that they had multiple peaks of spatially selective firing. We also detected DS units that were responsive specifically to novel object exploration, indicating that a subset of DS neurons were tuned to novelty during the NOR task. We have thus identified clear neurophysiological correlates for recognition within the DS, at the network and single-unit levels, strongly suggesting that it participates in encoding recognition-related signals.

## Introduction

Recognition refers to the ability to quickly determine whether an item (or event) is novel or has been experienced before. It is a fundamental feature of mammalian behavior and a prerequisite to store experiences into memory. Decades of research have shown that recognition memory can be functionally segregated into the processes of familiarity (referring to a vague feeling of knowing) and recollection (referring to the retrieval of fully formed episodes). Ample results from neurological and neuroimaging studies in human subjects, together with behavioral and neurophysiological data in animals (monkeys and rodents), indicate that the medial temporal lobe (MTL) plays a crucial role in supporting recognition memory (see Eichenbaum et al., 2007 for review). The MTL consists of the perirhinal cortex, the parahippocampal cortex (called postrhinal cortex in rodents), and the entorhinal cortex, as well as the hippocampus, which comprises the dentate gyrus, Ammon's horn, and the subiculum.

An influential “dual-process” model states that the parahippocampal and perirhinal cortices support familiarity, whereas the hippocampus is critical for episodic recollection (Eichenbaum et al., 1996; Yonelinas, 1999; Brown and Aggleton, 2001). In its strictest version, this model posits a complete functional segregation so that, for instance, selective damage to the hippocampus would result in impaired recollection but intact familiarity. Although the dual-process model has gained increasing experimental support (Eichenbaum et al., 2007), recent studies have challenged it and proposed an alternative model in which a single process encodes the recognized item based on its memory strength (see Shimamura, 2010 for review). Hence, a considerable debate has ensued about the neural code for recognition memory in the different regions of the MTL (Voss and Paller, 2010).

The subiculum represents an intriguing area of the MTL in which to examine the neural basis of recognition. The subiculum is typically described as an output structure of the hippocampus because it receives a massive input from CA1 and sends numerous projections to cortical and subcortical targets. As such, the subiculum is thought to be the last stage of hippocampal processing from which a highly processed episodic code is sent out to the neocortex (O'Mara, 2005). This view is strengthened by the presence of spatially selective place cells (Sharp, 1997; Anderson and O'Mara, 2004; Sharp, 2006) and vector-bound cells (Lever et al., 2009) in the subiculum, indicating that this structure is likely involved in processing a spatial code. However, the subiculum also receives extensive projections from the perirhinal cortex, which predominantly terminate in the proximal third of the dorsal subiculum (DS), closest to the CA1 border (Amaral and Lavenex, 2007). In fact, monosynaptic and reciprocal connections have been described between the DS and the perirhinal and postrhinal cortices (Naber et al., 1999; Witter et al., 2000). This connectivity pattern implies that the subiculum is part of a short functional loop with cortical areas that are crucially engaged in the neural processing of recognition memory. There is a scarcity of data to support this claim, but some studies suggest that the subiculum might play a role in stimulus recognition and memory processing that does not depend on the CA1 field (Galani et al., 1998; O'Mara, 2005; Potvin et al., 2010).

In rodents, recognition memory can be studied with a novel object recognition (NOR) task that takes advantage of the animal's spontaneous tendency to explore novelty, so it does not require extensive training (Ennaceur and Delacour, 1988; Bevins and Besheer, 2006). In this sense, the NOR task is well grounded ethologically as it reflects recognition behaviors that are naturally occurring in mammals. Crucially, it requires the intact memory of a previously experienced object, concurrently with the perception of a novel object. Previous studies using the NOR task have highlighted the role of the perirhinal cortex (Brown and Aggleton, 2001) and have produced mixed results with regards to the contribution of the hippocampus (Hammond et al., 2004; Nyberg, 2005; Winters and Bussey, 2005).

In this study, we hypothesized that the subiculum may be a crucial substrate for recognition memory within the rodent brain. We tested this idea by monitoring network oscillations in freely behaving mice while they were introduced to both environmental novelty and object novelty in the context of the NOR task. We were particularly interested in how oscillations in the theta range (4–12 Hz) might be modulated as mice underwent the different stages of the task. Theta oscillations depend on ongoing behavior and are typically present during rapid eye movement sleep and during various types of locomotor activities described as voluntary, preparatory, orienting, or exploratory (Vanderwolf, 1969). Since these oscillations are known to reflect the behavioral state of the animal (Buzsáki, 2006; O'Keefe, 2007), *in vivo* recordings from freely behaving animals offer a strong link between complex behaviors and their underlying neural bases (Mithra and Bokil, 2008). An emerging body of evidence has highlighted the connection between specific neuronal circuits and cognitive behaviors (Montgomery and Buzsáki, 2007; Herry et al., 2008). Thus, we wanted to study how the naturally occurring behavior of bias toward novelty could be encoded within the mouse brain. Since other MTL structures garner the majority of the attention in the study of recognition memory, we wanted to examine whether the DS was also a potentially significant locus in the neural processing of novelty.

## Materials and methods

### Animals

The Feinstein Institute Animal Care and Use Committee approved all animal procedures. Female BALB/cJ mice (Jackson Labs) were chosen because our pilot studies indicated that females of this strain maintained high levels of exploration throughout the NOR task, thus optimizing data collection. Mice (*n* = 12) were kept on a reverse schedule of 12 h of darkness (08:00–20:00) and 12 h of light, with *ad libitum* access to food and water. About a week before surgery, mice were handled in daily sessions of 5–10 min. Both the handling and the subsequent experiments were conducted during the dark period of their circadian cycle.

### Electrode array implantation

**Figure 1:**
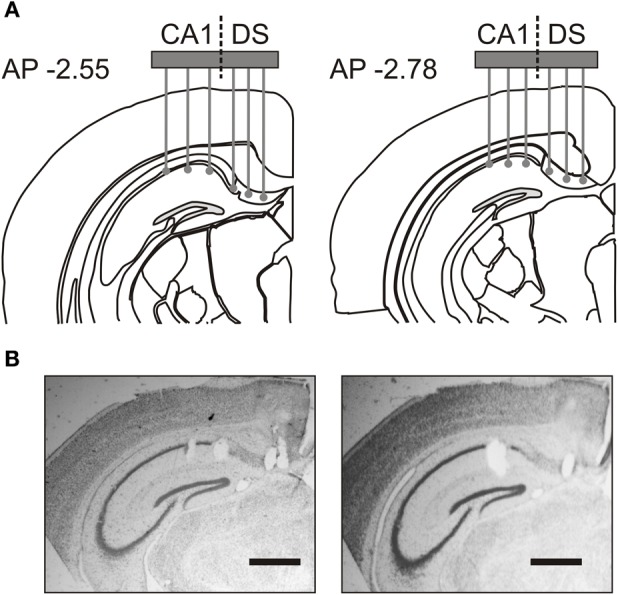
**Placement of recording electrode array. (A)** Coronal schematics indicate electrode recording positions in the most anterior (AP –2.55) and most posterior (AP –2.78) placements across implanted mice. **(B)** Representative Nissl-stained sections, from the most anterior placement in two animals, show electrode tip lesions in hippocampal area CA1 and dorsal subiculum (DS). Scale bar, 500 μm.

Young adult mice weighing 24–32 g were anaesthetized using a mixture (2.5 ml kg^−1^) of ketamine (50 mg ml^−1^), xylazine (2.6 mg ml^−1^), and acepromazine (0.5 mg ml^−1^) and were placed in a stereotaxic frame. Using a surgical drill (Foredom Electric, Bethel, CT), a craniotomy was performed in the right hemisphere centered at coordinates −2.7 mm AP, 1.0 mm LM. A hole was drilled for a ground screw posterior to lambda, above the cerebellum, and a second hole was drilled anterior to bregma for a skull screw that would provide a support point for the microdrive assembly. The microdrive contained an electrode array of 50-μM nickel-chromium wires (Stableohm) or 13-μM platinum-iridium tetrodes (California Fine Wire, Grover Beach, CA) that could be vertically lowered into the DS and the dorsal CA1 field (Figure 1A). Immediately following the craniotomy, implanted mice were monitored until they were fully awake and ambulating. Out of the 12 attempted surgeries, 3 mice did not survive the surgical procedure. The surviving mice were allowed 7 days to recover, handled again, and acclimated to the experimental room. The array was lowered 960-μM on the day of the surgery. On the first day of recording, the array was lowered in 70-μM steps until the electrode tips reached their final recording depth at the level of the *stratum pyramidale* of CA1 and DS (Figure 1A).

### Familiarization to the chamber before the task

The apparatus consisted of a chamber with a square base (30 cm on the side) and 80-cm high walls made of white polyvinyl chloride. The floor was covered with bedding that was similar to the bedding used in the home cage. A light bulb (50 W) of orange–red hue illuminated the chamber from above. An infrared-sensitive camera (Panasonic) was mounted above the chamber and connected to the input of a video capture card (Data Translation DT3120, Marlboro, MA) inside a PC running behavioral software (AnyMaze, Stoelting Co., Wood Dale, IL) that recorded the animal's behavior at 30 frames/s during all experimental trials. The implanted mouse was transported inside the home cage into the darkened experimental room. Immediately after connecting the microdrive to the recording system, the animal was placed in the empty chamber (without any objects) for 15 min. Each mouse had four familiarization sessions to insure full acclimation to the context.

### Novel object recognition task

The NOR task consisted of a sample trial (5 min), followed by a delay period (10 min), and a choice trial (5 min). This sequence was repeated at least twice per day and at least on three consecutive days for each mouse. As in the familiarization sessions, the mouse was transported into the darkened experimental room, connected to the recording system, and immediately placed in the experimental chamber. For the sample trial, mice explored the chamber in the presence of two identical objects. Following the delay period in a highly habituated holding chamber, mice were returned to the experimental chamber for a choice trial in which they explored a triplicate copy of the sample object and a novel object. In order to minimize olfactory cues, the objects were cleaned between each trial with a 70% ethanol solution and allowed to air dry before being returned to the experimental chamber. In a separate study, we determined the relative salience of a large group of objects and selected only objects that were comparable in salience for this study.

We used behavioral tracking software (AnyMaze) to obtain the onset and duration of each epoch of object exploration, which was strictly defined as a period when the animal's snout was in close proximity (<1 cm) to the object's periphery. We used the number of visits and the times spent exploring each object on sample and choice trials for statistical comparisons. For sample trials, an exploration ratio was defined as “the time exploring the right object divided by the sum of the times exploring the right and left objects”. For choice trials, a novelty ratio was defined as “the time exploring the novel object over the sum of the times exploring the novel and familiar objects”.

### Neuronal recordings in freely behaving mice

We recorded neural activity via a unitary gain headstage preamplifier (HS-18, Neuralynx, Bozeman, MT). Local field potentials (LFPs) were acquired at a sampling rate of 3 kHz and band-pass filtered (0.1–500 Hz) by a Lynx-8 programmable amplifier (Neuralynx) on an Intel Core 2 Duo personal computer running acquisition software (Cheetah, Neuralynx). The same recording system was used to acquire single units at a sampling rate of 30 kHz and a band-pass filter between 500 Hz and 9 kHz. Continuous LFP and single unit data were analyzed using NeuroExplorer version 3 (NeuroExplorer, Littleton, MA), OfflineLineSorter (Plexon, Dallas, TX), and Spike2 (Cambridge Electronic Design, Cambridge, UK) software packages. Two light-emitting diodes on the implanted electronic interface board were used for tracking the location of the mouse in space at 30 frames/s by the acquisition software (Cheetah, Neuralynx).

The final electrode positions were marked with electrolytic lesions (0.1 mA for 10 s) after the final recording session. Mice were then sacrificed and their brain tissue was processed for a Prussian Blue reaction and Nissl staining (Figure 1B). Recording sites were reconstructed using a combination of electrophysiological markers, microdrive movement, and post-mortem histology. Out of the nine experimental animals, we excluded the data from two mice because some of the electrode positions were outside of the target zones in CA1 or DS. Therefore, only the results from seven implanted mice were used for final analysis.

### Analysis of local field potentials

LFPs were continuously recorded during the familiarization sessions and the NOR task. To analyze the familiarization data, continuous LFP epochs were used to generate power spectral density (PSD) plots for each session. For the NOR task, LFP epochs were extracted selectively for periods of object exploration and grouped according to the particular object visited; “left” and “right” in the sample phase; “familiar” and “novel” in the choice phase. We then obtained PSD plots for each of these LFP groupings and, from the PSD plots, calculated the average power by taking the integral of the PSD in the 4–12 Hz range. Therefore, for each NOR session, we computed the theta power for “left” and “right” in the sample phase; and “familiar” and “novel” in the choice phase. In order to compare across sessions and animals, we normalized the data with the use of a “theta power ratio,” which for the sample phase was defined as “right theta power divided by left theta power,” and for the choice phase was defined as “novel theta power over familiar theta power.”

### Analysis of single unit activity

Putative single units in CA1 and DS were amplitude thresholded and then sorted with principal component analysis followed by manual cluster cutting (OfflineLineSorter, Plexon). Spike-related parameters such as spike width, amplitude, shape, timing, and rate were used to subsequently categorize units as principal neurons or interneurons. We calculated the theta modulation index (TMI) for the recorded units, according to Cacucci et al. (2004). The TMI was derived by computing the theta modulation trough (TMT, mean autocorrelogram value at 50–70 ms) and the theta modulation peak (TMP, mean autocorrelogram value at 100–140 ms), and then taking their difference over their sum, so that TMI = (TMT − TMP)/(TMT + TMP). We constructed firing rate maps by calculating the total number of spikes for each pixel and then dividing by the dwell time for a particular session. Place cell field size was calculated as at least eight contiguous pixels that shared an edge and were at least 20% of the peak-firing rate for that unit. For units displaying more than one place field, the place field size was computed as the sum of the existing fields (Brontons-Mas et al., 2010).

### Statistical analysis

Data are presented as mean ± SEM, as indicated. We used factorial ANOVA, repeated measures ANOVA, and Student's *t*-test to examine statistical significance, which was defined as *P* < 0.05.

## Results

### Network oscillations were lower in power in DS during familiarization

**Figure 2:**
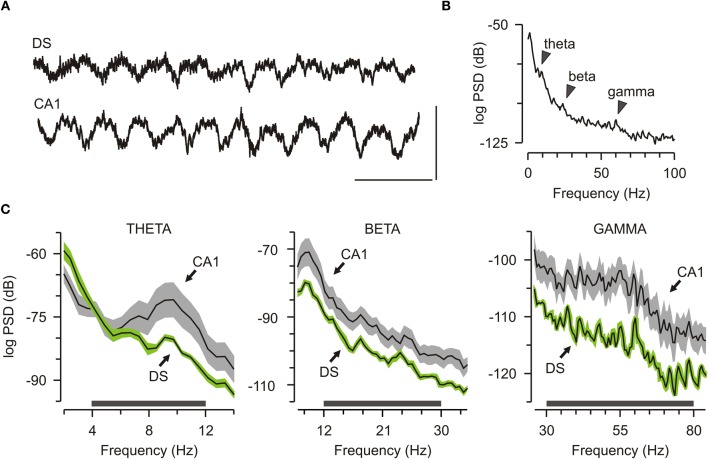
**Robust network oscillations in the dorsal subiculum. (A)** Traces showing local field potential from the dorsal subiculum (DS) and CA1. Scale bar, 200 ms (x axis), 1 mV (y axis). **(B)** Representative graph of the power spectral density (PSD) from a DS electrode taken from a 15-min familiarization session. Arrows mark peaks for theta, beta, and gamma bands. **(C)** Grouped data (mean ± SEM) across the initial familiarization sessions (*n* = 7) for theta (4–12 Hz), beta (12–30 Hz), and gamma (30–80 Hz) show that DS has lower power than CA1 across these bands.

We monitored LFPs within DS and CA1 of freely behaving mice (*n* = 7), with the rationale that network activity offered a strong link between complex behaviors and their underlying neural bases (Mithra and Bokil, 2008). There was marked oscillatory activity in DS and CA1 during familiarization to the chamber, before objects were introduced (Figure 2A). Power spectral density (PSD) plots revealed clear peaks on the theta band (4–12 Hz), beta band (12–30 Hz), and gamma band (30–80 Hz) for the DS oscillations (Figure 2B), which were similar to the peaks previously reported for CA1 (Buzsáki, 2006; O'Keefe, 2007). Closer comparison between the two structures revealed that the theta peak had lower energy in DS than CA1, and similar signatures were found for the beta and the gamma components (Figure 2C).

### CA1-theta decreased during familiarization to the experimental chamber

**Figure 3:**
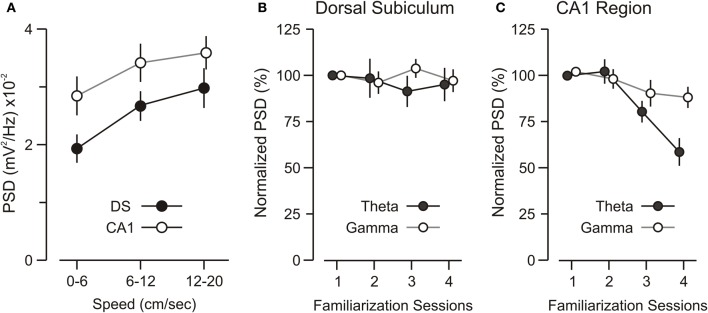
**Theta and gamma power during familiarization. (A)** Graph showing that theta power increases over running speed and that theta power differences between the DS and CA1 remain across speeds. **(B)** Graph of theta and gamma power in DS for sequential 15-min sessions of familiarization to the experimental chamber (without objects). DS theta and gamma are unaffected by familiarization over repeated sessions. **(C)** CA1 theta power decreases significantly over familiarization sessions, as demonstrated by ANOVA [*F*_(3, 18)_ = 28.85, *p* < 0.001]. *Post-hoc* paired comparisons reveal that the theta band power during session 1 is significantly higher than session 3 and session 4 (*p* < 0.005, *t*-test). CA1 gamma power is unchanged across sessions. All values represent mean ± SEM.

It is known that CA1-theta magnitude changes with running speed (Vanderwolf, 1969), thus we parsed the network recordings obtained during familiarization to the experimental chamber into speed segments (0–6, 6–12, 12–20 cm/s) and analyzed the theta power for each segment. We found that both DS-theta and CA1-theta power showed a clear increase with speed (Figure 3A). ANOVA, with speed as the repeated measure, revealed a significant interaction between speed and theta power [ANOVA, *F*_(2, 22)_ = 37.96, *p* < 0.0001].

Moreover, we found that CA1-theta power decreased significantly as the mice were familiarized to the experimental chamber, without objects (Figure 3C) [ANOVA, *F*_(3, 18)_ = 28.85, *p* < 0.001]. This decrease could not be explained by a decrease in the mean speed over sessions. In fact, the mean speed did not differ significantly across sessions [ANOVA, *F*_(3, 18)_ = 3.43, *p* = 0.65], which was not surprising because BALB/cJ mice are known to maintain a high level of locomotion and rarely stop for sustained periods of time (Tang et al., 2002; Lepicard et al., 2006). Rather, this CA1-theta power decrease was likely due to lower levels of cholinergic drive from the septum as the novelty of the experimental chamber decreased over repeated exposures (Givens and Olton, 1995; Markowska et al., 1995; Giovannini et al., 2001). Notably, the theta power in DS remained unchanged over familiarization sessions (Figure 3B) [ANOVA, *F*_(3, 18)_ = 4.06, *p* = 0.69], pointing to a fundamental difference in theta activity between DS and CA1. Finally, analysis of gamma-band oscillations revealed that gamma power in the DS and CA1 was unaltered across familiarization sessions.

### Mice showed a robust bias toward novel objects

**Figure 4:**
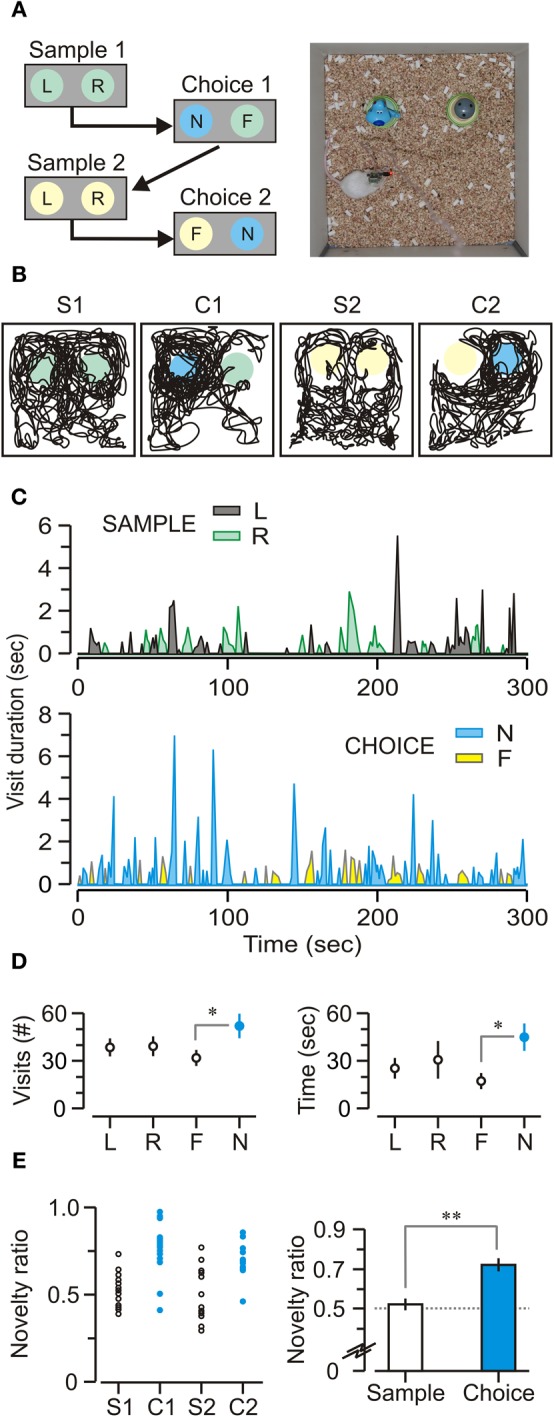
**Novel object recognition task. (A)** *Left*, schematic of the daily protocol comprising two phases of sample (5 min) and choice (5 min), separated by 10-min delays (represented by arrows). Abbreviations, F, familiar object; L, left object; N, novel object; R, right object. *Right*, top view of an implanted, freely behaving mouse performing a choice trial within the square chamber (30 cm on the side). **(B)** Track plots for a mouse during sample and choice phases that are labeled S1 (first sample trial), C1 (first choice trial), S2 (second sample trial), and C2 (second choice trial). Circles represent locations of the objects; colors, as in **A**. **(C)** Sample and choice trials showing the duration and frequency of visits over representative trials from a single implanted mouse. Abbreviations, as in **A**. **(D)** *Left*, number of visits (mean ± SEM) across trials showing that, on choice trials, mice visited the novel object (N) significantly more than the familiar one (F). *Right*, duration of visits (mean ± SEM) across trials showing the same pattern; on choice trials, mice spend a significantly longer time visiting the novel object. **(E)** *Left*, novelty ratios plotted across all sample (S1, *n* = 19, S2, *n* = 19) and all choice trials (C1, *n* = 16, C2, *n* = 14) showing that mice have a robust novelty bias on choice trials (C1 and C2). *Right*, novelty ratios (mean ± SEM) for all mice (*n* = 7). ^*^p < 0.005; ^**^p < 0.001 (*t*-test).

After the mice were habituated to the experimental chamber (without objects) in multiple familiarization sessions, they were exposed to sample and choice trials with 10-min delays between trials (Figure 4A). Animals showed a clear bias toward novel objects, indicating that their natural performance was unaffected by the implanted recording headstage (Figures 4B,C). On sample trials, mice visited the objects similar number of times (Figure 4D, left) [visits to left = 38.4 ± 3.2, visits to right = 39.1 ± 3.9 per trial; *t*-test, *t* = 0.14, *p* = 0.9] and spent comparable total time across trials exploring each object (Figure 4D, right) [total time left = 25.5 ± 3.8, total time right = 30.8 ± 9.4 s per trial; *t*-test, *t* = 0.52, *p* = 0.6]. On choice trials, mice visited the novel object significantly more (Figure 4D, left) [number of visits to familiar = 31.7 ± 2.5, number of visits to novel = 51.9 ± 5.3 per trial; *t*-test, *t* = 5.43, *p* < 0.005] and also spent more total time exploring the novel object (Figure 4D, right) [total time familiar = 17.4 ± 2.5, total time novel = 45.0 ± 6.2 s per trial; *t*-test, *t* = 4.76, *p* < 0.005]. Moreover, it was clear that animals preferred to explore the novel object during choice trials (Figure 4E) [exploration ratio = 0.54 ± 0.02, novelty ratio = 0.72 ± 0.03, *t*-test, *t* = 5.64, *p* < 0.001].

### Theta power in DS was modulated by object novelty

**Figure 5:**
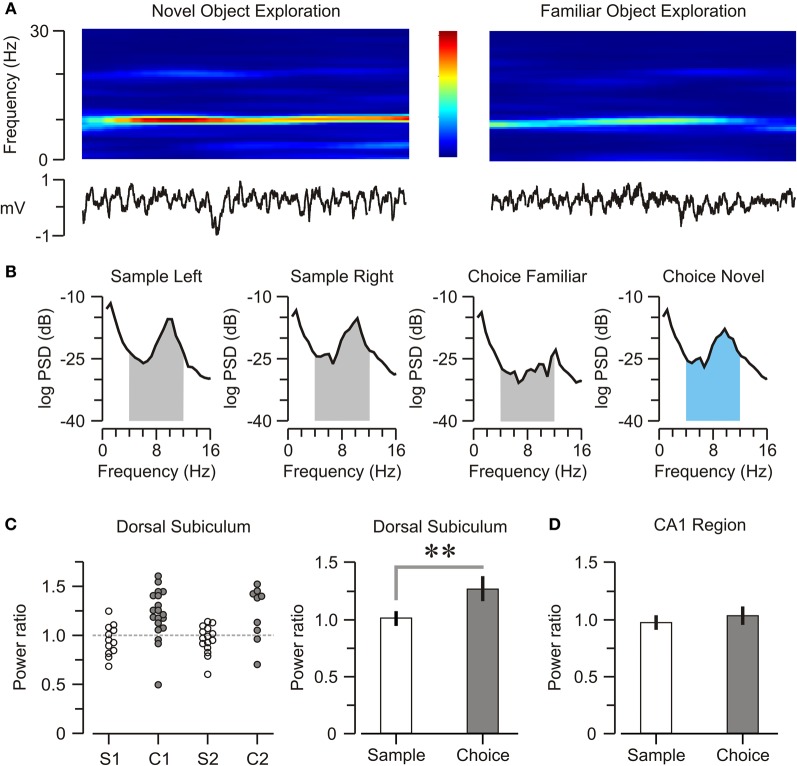
**Subicular encoding of object novelty. (A)** *Top*, spectrogram showing spectral density for a single representative DS electrode during 2-s episodes of novel and familiar object exploration. The color scale (middle panel) varies from red (high power) to blue (low power), within 0–30 Hz frequency range. *Bottom*, unfiltered neural activity during the 2-s episodes. **(B)** Representative PSD plots taken only during periods of object exploration. The shaded areas under the curves represent the respective theta bands. On choice trials, DS-theta power increases specifically when mice explore the novel object (graph with theta band indicated in blue). **(C)** *Left*, graph displaying individual theta power ratios in the DS during sample (S1, *n* = 19, S2, *n* = 19) and choice (C1, *n* = 16, C2, *n* = 14) trials across all mice. *Right*, on average, the theta power ratio (mean ± SEM) is elevated on choice trials. **(D)** Graph showing that, in CA1, there is no difference in theta power ratios (mean ± SEM) between sample and choice trials. ^**^p < 0.001 (*t*-test).

Oscillatory episodes were obvious in DS and CA1 across the NOR task, but examination of spectrograms suggested that theta power in DS was elevated when mice explored novel objects (Figure 5A). We isolated LFPs only for epochs of object exploration within a trial, built PSD plots for those epochs (Figure 5B) and calculated the DS-theta power. On sample trials, DS-theta power was equally elevated when mice inspected both objects [right = 245.9 ± 8.7, left = 235.8 ± 7.9 dB^*^Hz, *n* = 42; *t*-test, *t* = 0.86, *p* = 0.39]. Remarkably, on choice trials, DS-theta power was significantly higher during novel object exploration [novel = 253.2 ± 7.6, familiar = 199.2 ± 7.9 dB^*^Hz, *n* = 26; *t*-test, *t* = 4.69, *p* < 0.001]. We also calculated a “theta power ratio” (see Materials and Methods for definition) and found it was significantly higher on choice vs. sample trials (Figure 5C) [choice = 1.27 ± 0.08, sample = 1.04 ± 0.06, *n* = 68; *t*-test, *t* = 4.4, *p* < 0.005]. In contrast, theta power within CA1 was not correlated with object novelty and had comparable theta power ratios on both trial types (Figure 5D) [choice = 1.03 ± 0.07, sample = 0.97 ± 0.05, *n* = 68; *t*-test, *t* = 0.29, *p* = 0.77].

### CA1–DS coherence was higher during contextual exploration

**Figure 6:**
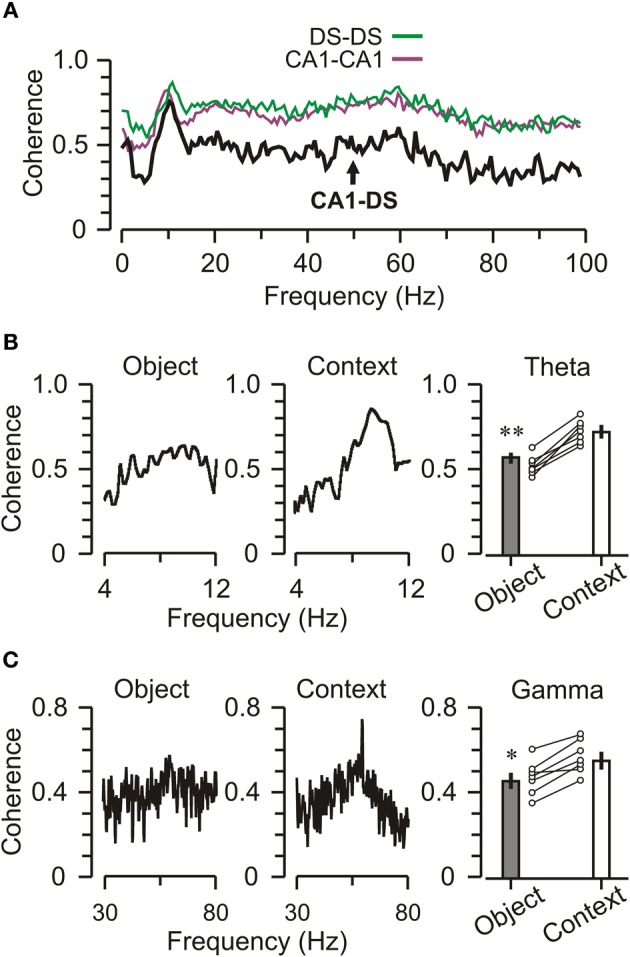
**Decreased DS-CA1 theta coherence during object exploration. (A)** Graph of coherence values across oscillation frequencies for a 5-min trial when the mouse is not exploring objects. Intra-regional coherences (CA1-CA1, DS-DS) are higher than inter-regional coherence (DS-CA1), particularly in the gamma band. To generate these graphs, a CA1 channel was referenced to another electrode within CA1 (CA1-CA1, purple) or the DS (CA1-DS, black), or a DS channel was referenced to another DS channel (DS-DS, green). **(B)** *Left*, plot for a representative DS-CA1 pair showing modest coherence in the theta band during object exploration. *Middle*, plot shows that theta band coherence increases during context exploration. *Right*, graph showing that the effect is significant across CA1-DS pairs, circles represent the mean coherence for CA1-DS pairs from individual mice (*n* = 7) and bar graphs show group means ± SEM for the theta band. **(C)** Plots for a single DS-CA1 pair (left and middle) and grouped means across mice (*n* = 7, right) showing a similar, but less pronounced, pattern of elevated coherence in the gamma band during context exploration. ^*^p < 0.05; ^**^p < 0.005 (*t*-test).

To investigate the coordination of neural activity among regions, we measured DS–CA1 coherence (Montgomery and Buzsáki, 2007; Mithra and Bokil, 2008) across spatially separated electrodes (Figure 6A). Frequency-specific correlations between oscillations at different electrode sites, allow for the quantification of signaling between brain regions (Ruchkin et al., 2003). Theta coherence is typically higher between homotypic locations (e.g., CA1–CA1) across hemispheres than between less distant longitudinal sites within the same hemisphere, illustrating that theta varies as a function of connectivity rather than physical distance within the brain (Sabolek et al., 2009). We found, not surprisingly, a high level of intra-structural coherence across frequencies, but especially within the theta and gamma bands. In an effort to link inter-structural coherence with ongoing behavior, we separated epochs of object exploration from those of contextual exploration (periods when the animal was exploring the context during a trial). When we parsed the LFPs according to these behavioral contingencies, we found a clear coherence peak in the theta band when mice explored the context, which was noticeably absent when mice explored objects (Figure 6B). Statistical analysis indicated that DS–CA1 coherence was significantly higher for contextual vs. object exploration (Figure 6B, right) [theta band, *t*-test, *t* = 4.62, *p* < 0.001]. A similar pattern occurred for the gamma band (Figure 6C), which also showed enhanced DS–CA1 coherence for contextual vs. object exploration (Figure 6C, right) [gamma band, *t*-test, *t* = 3.95, *p* < 0.005]. Interestingly, DS–CA1 coherence was also noticeably decreased in the delta band, defined as 1–4 Hz, which may have consequences for working memory (Fujisawa and Buzsáki, 2011) but was not examined further here.

### Subicular units displayed weak phase locking to theta rhythm

**Figure 7:**
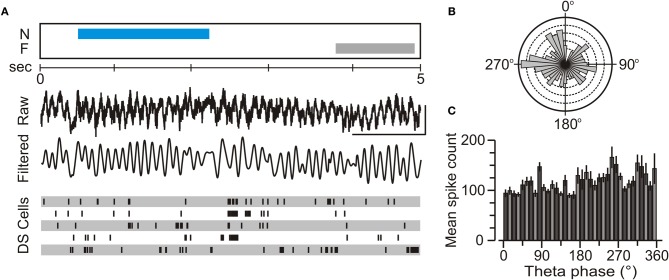
**Weak modulation of subicular unit firing by theta oscillations. (A)** Representative 5-s snapshot during a choice trial of the NOR task showing, from top to bottom, an epoch of novel object exploration (N), and epoch of familiar object exploration (F), the unfiltered neural activity (Raw), the band-pass (4–12 Hz) neural activity (Filtered), and raster plots for the firing of five single DS cells. **(B)** Rose diagram showing the firing of a DS unit with respect to theta phase, collected during a 5-min choice trial. In this diagram, 90° is the theta trough and 270° is the theta peak. This unit exhibits a highly dispersed theta phase distribution with a mean of 195 ± 118° (Rayleigh test; *Z* = 0.12, *p* = 0.89), indicating poor phase locking to the theta oscillation. **(C)** Distribution of spike times across theta phases (mean ± SEM) for a representative DS cell over multiple trials indicates weak phase locking.

Since the phase at which a neuron fires in relation to the ongoing theta oscillation is known to be important for spatial coding in CA1 (O'Keefe and Recce, 1993; Jensen and Lisman, 2000), we examined the phase relation between subicular firing and theta rhythm, focusing on the choice phase of the NOR task. Cursory examination of neuronal spiking aligned to the ongoing theta waves (and the behavioral contingencies) did not reveal any obvious theta-modulated spiking (Figure 7A). Therefore, we decided to use a quantitative parameter, termed TMI (Cacucci et al., 2004), to examine this issue further. We measured the TMI from DS principal neurons (*n* = 28) as well as CA1 pyramidal units (*n* = 51) that were concurrently recorded during the choice phase of the NOR task (see below for classification criteria). We found that the DS units had a mean TMI of 0.12 ± 0.05, which was significantly lower than the TMI of 0.25 ± 0.03 for CA1 place cells (*t*-test, *t* = 4.1, *p* < 0.001). Additionally, the subicular neurons did not exhibit strong phase locking to the prevailing theta oscillation, as shown by their theta phase distribution plots (Figures 7B,C).

### A subset of DS units responded to object novelty

**Figure 8:**
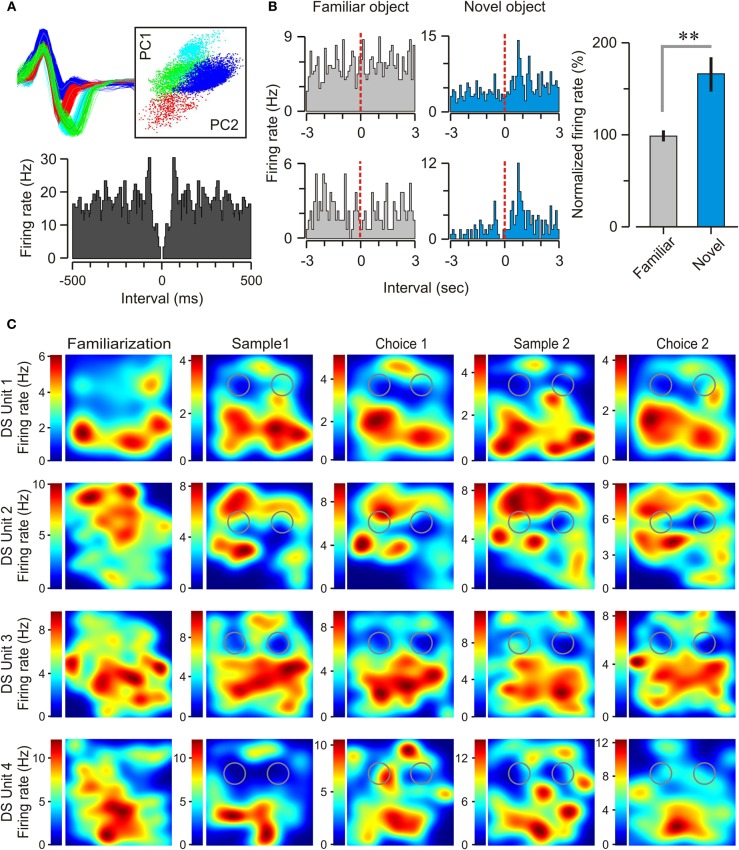
**Spatial selectivity and object responsiveness of dorsal subicular neurons. (A)** *Top left*, action potentials from three subicular neurons are shown in red, blue, and green. *Top right*, the spikes are isolated by principal component clustering, of which two projections (labeled PC1 and PC2) are shown. *Bottom*, autocorrelogram for a principal neuron recorded during a choice trial. **(B)** *Left*, peri-event histograms for two novelty-responsive DS neurons, one on each row, during exploration of familiar and novel objects. Dashed lines indicate the onset of object exploration. *Right*, Plot of the normalized firing rate (mean ± SEM), 500–1000 ms after the start of exploration of familiar and novel objects for this subset of DS neurons. ^**^p < 0.005 (*t*-test). **(C)** Firing rate maps of four DS neurons during the sequential stages of the NOR task showing broad place fields with several peaks of firing within the behavioral context. In these top views of the square chamber (30 cm on the side), gray circles indicate the locations of the objects. The color scales (at left of each map) specify the firing rates (spikes per sec) for the units. It is clear that subicular place cells exhibit spatial selectivity that does not appear to be modulated by the presence of objects nor their degree of novelty.

As already reported by others (Barnes et al., 1990; Sharp and Green, 1994; Gigg et al., 2000; Anderson and O'Mara, 2004; Brontons-Mas et al., 2010), it was significantly harder to record well-isolated DS units, as compared to concomitantly recorded CA1 units. Over 96 recording sessions, we obtained a total of 38 DS units, 28 of which were identified as principal cells and 10 as interneurons on the basis of their firing rates, autocorrelograms, and spike widths (Figure 8A, Table 1). By comparison, we recorded a total of 72 CA1 units, 51 of which were classified as pyramidal cells and 21 as interneurons. We found that the majority of the subicular neurons (32 of 38, ~84%) were not responsive to objects, meaning that they did not change their firing rate (increase or decrease) as a function of the mouse exploring either a novel or a familiar object. However, we were able to isolate a subset of DS principal neurons (6 of 28, 21.4%) that were specifically modulated during the exploration of novel objects. Figure 8B shows representative examples of these novelty-responsive subicular cells and their firing rates in relation to approaching a novel object or a familiar object. There was a marked increase in spiking after the onset of novel object exploration, which peaked at 796.4 ms after onset, and which was completely absent during the exploration of familiar objects. Analysis of multiple epochs showed that this was a statistically robust phenomenon. We examined the firing in the interval of 500–1000 ms after the start of object exploration, and found that the novelty-responsive DS cells had a significantly higher firing rate for novel objects as compared to familiar objects (Figure 8B, right) [novel = 165 ± 19%, familiar = 98 ± 6%, *n* = 211 visits; *t*-test, *t* = 3.46, *p* < 0.005]. These particular DS neurons may be critical within a recognition memory neural system, given their heightened responsiveness to novel stimuli.

**Table 1:** **Properties of single units in DS and CA1**.

**Cell type**	***N***	**Firing rate (Hz)**	**Spike height (μV)**	**Spike width (msec)**
DS principal	28	1.24 ± 0.25	238.5 ± 32.7	0.69 ± 0.1
DS interneuron	10	7.9 ± 1.8	198.1 ± 39.4	0.20 ± 0.09
CA1 principal	51	1.08 ± 0.21	252.6 ± 35.1	0.58 ± 0.12
CA1 interneuron	21	14.5 ± 3.9	215.8 ± 20.1	0.16 ± 0.31

### Place cells in DS were multi-peaked and broader than CA1 place cells

We found that subicular neurons in the mouse had spatial specificity, but with relatively low spatial tuning (Figure 8C) in comparison to CA1 place cells. The mean size of the CA1 fields was 297 ± 24.4 cm^2^, which on average occupied 33% of the experimental chamber. On the other hand, the mean size of DS fields was 443 ± 23.9 cm^2^, and covered 49.2% of the chamber. Statistically, DS place cells were significantly larger when compared to CA1 place cells (*t*-test, *t* = 4.26, *p* < 0.005). Additionally, DS place cells expressed significantly more fields than CA1 place cells; DS place cells had an average of 2.39 ± 0.20 fields compared to CA1 cells with an average of 1.38 ± 0.10 fields (*t*-test, *t* = 4.58, *p* < 0.0001).

## Discussion

We have used spontaneous bias toward novelty, a robust behavioral response in mammals, to examine the contribution of the DS to the neural substrate of recognition memory. We found that theta-band oscillations in the DS were clearly modulated by object novelty. We propose that the enhanced DS-theta power when mice explore novel vs. familiar objects reflects a neural signature for object novelty (Figure 5C). Another crucial finding was the occurrence of heightened coherence of theta and gamma oscillations between DS and CA1 when mice were moving through the environment without exploring objects (Figures 6B,C). We think this reflects an increased interaction between the DS and CA1 during episodes of spatial orientation. These results concur with an emerging body of evidence that highlights coherent interactions between specific brain regions as markers for cognitive tasks (Seidenbecher et al., 2003; Buzsáki, 2006; Montgomery and Buzsáki, 2007; Herry et al., 2008; Düzel et al., 2010; Shirvalkar et al., 2010; Fujisawa and Buzsáki, 2011).

Changes in theta power during behavior have been linked to some types of learning, both in rodents and humans (Caplan et al., 2003; Wyble et al., 2004; Paz et al., 2008). Our study shows that, in the choice phase of the NOR task, there was a significant elevation of DS-theta power as the animal explored novel objects (Figure 5C). Meanwhile, DS-theta power was comparably high during the exploration of both objects in sample trials. This pattern of results can be explained by considering that during the sample phase both objects are equally novel to the mouse. Therefore, DS-theta power for the “left” and the “right” objects might be conceptualized as reflecting a subicular network response to novelty, just as it occurs with novel objects in the choice trials. Thus, our study has identified an electrophysiological signature within the DS that closely correlates with exploration of novel objects in the NOR task.

Although we postulate that the modulation of DS-theta power correlates with object recognition, it remains possible that DS-theta might be altered by subtle differences in movement (running speed) or sensorimotor processes. With respect to movement, we think the enhanced DS-theta power during bouts of novel object exploration cannot be dismissed as an epiphenomenon (resulting from changes in movement or running speed) because the increased power occurred when mice were consistently motionless or moving slowly (i.e., 0–6 cm/s) as they explored novel objects. Furthermore, the decreased DS-theta power observed during exploration of familiar objects may reflect a switch of the relevant networks to a lower state of attention, and thus it may represent a possible neural signal for familiarity. It is known that neurons in the hippocampus and perirhinal cortex respond in fundamentally different ways to the repetition of a stimulus (Brown et al., 2010) and the differential response we report here, measured at the network level, might be evidence of a similar process within the DS. Regarding sensorimotor processing, hippocampal theta oscillations are well known to correlate with sniffing (Macrides, 1975; Kay, 2005). While we attempted to minimize any potential olfactory cues by cleaning the objects between all trials, we cannot exclude the possibility that novel objects triggered a fundamentally distinct pattern of sniffing and exploration (compared to familiar objects), and that these behavioral differences influenced the DS-theta power modulation we observed. Additional in-depth analyses are needed in order to dissect these potential sensorimotor contributions.

This study makes use of a technique that might be termed “behavioral clamping” because spontaneous, short-lived behavioral events that occur during the task (i.e., object exploration bouts) are used for selecting, with millisecond precision, the oscillatory networks patterns within the hippocampus that will be subjected to further analysis. This is a powerful technique but it is also important to realize that by clamping the neural activity through precisely timed behavioral contingencies; we are only taking snapshots of the ongoing neural processes. Although this allows us a glimpse into the neural basis of these behaviors, one must be aware that the DS and CA1 are only a part of a larger and more extensive neural system.

In contrast to the DS, CA1-theta power was stable during novel and familiar object exploration, a result that is consistent with a previous study in the rat (Manns et al., 2007). However, CA1-theta power decreased over repeated exposures to the same environment (Figure 3C). This CA1-specific effect remained even after controlling for running speed during familiarization sessions. We think it is unlikely that any changes in CA1 theta were due to drifting of the electrodes from *stratum pyramidale* into another layer, which would result in a theta amplitude change. This assumption was supported by inspection of the electrode tips after the recordings and the fact that DS-theta remained completely unchanged during the familiarization sessions, thus providing an internal control for the CA1 recordings. Neurophysiological signals for environmental novelty have been studied previously in CA1 (Fontani et al., 1984; Jeewajee et al., 2008; Lever et al., 2010), which has been proposed as a substrate for mismatch detection (Honey et al., 1998; Vinogradova, 2001). While we found that CA1-theta power declined over multiple familiarization sessions, we did not observe the significant shift in theta peak frequency (lower frequency on novel and unexpected environments) that was reported in rats (Jeewajee et al., 2008). However, it should be noted that we did not change environments over multiple sessions in order to explicitly test for this effect.

We observed heightened theta–gamma coherence between DS and CA1 when mice explored the chamber's context rather than the objects (Figure 6). This result suggests that the DS is capable of segregating the spatial code emerging from CA1 from the novelty code, presumably emerging from the perirhinal cortex. Since theta and gamma provide a timing mechanism to coordinate the activity of subsets of neurons, the increased DS–CA1 coherence during contextual examination may reflect the formation of transient cell assemblies in CA1 and DS that encode the spatial code. Indeed, CA1–subicular synapses are capable of undergoing long-term potentiation (Commins et al., 1999; Kokaia, 2000; O'Mara et al., 2000; Huang and Kandel, 2005), which may play a role in the establishment of CA1-DS cell assemblies.

While we focused our study on the network oscillations of the DS, we also recorded DS single units (*n* = 38) and, concomitantly, CA1 single units (*n* = 51). A vast literature is available on CA1 pyramidal neurons recorded on freely behaving rodents, but there are relatively few studies of subicular neurons and their firing properties in similar conditions (Barnes et al., 1990; Sharp and Green, 1994; Gigg et al., 2000; Anderson and O'Mara, 2004; Brontons-Mas et al., 2010). Notably, we discovered a small population of DS neurons that appeared to be tuned specifically to novel object exploration (Figure 8B). We also identified DS neurons with broad spatial specificity, expressed as multiple place fields (Figure 8C). Subicular place cells have been reported previously in the rat (Sharp and Green, 1994; Anderson and O'Mara, 2004). Our study did not test the mice in different environmental contexts to assess whether DS place cells generated unique place fields for each context (Sharp and Green, 1994). With the discovery of grid cells in the entorhinal cortex (Hafting et al., 2005), the evidence of broad, multiple-peaked subicular place cells should be considered within a model of spatial processing that occurs within the whole hippocampal formation.

Our results have direct implications for the subject of the global function of the subiculum. A commonly held idea is that this region participates in late stages of spatial processing within the hippocampal formation, a view that is supported by the presence of subicular place cells (Sharp, 1997; Anderson and O'Mara, 2004; Sharp, 2006) and vector-bound cells (Lever et al., 2009). Moreover, this view typically assigns a secondary role to network-level processes, such as subicular theta and gamma, as clocking mechanisms. Our data suggests a more complex scenario in which the subiculum participates, at the cellular and network levels, in both spatial coding and recognition-related coding. Previous models of subicular function have not considered such a dual coding system, and the manner in which these codes might coexist within the subiculum. Interestingly, the seminal theory of cognitive mapping by O'Keefe and Nadel (1978) included the possibility that their proposed hippocampus-based spatial mapping system would be interrupted when an animal encountered novel items or events. A different system would take temporary control over behavior and would trigger exploration of the novel item (p. 242, O'Keefe and Nadel, 1978). These early ideas are congruent with our results of CA1–DS coherence and point to a mechanism in which the subiculum functions by *segregating* spatial processing from recognition signaling. In other words, as the animal navigates in the environment, the subiculum would encode spatial signals (such as coding of distance relations between objects and arena wall or distant information) in conjunction with CA1, leading to high CA1–DS coherence in the theta and gamma range. Conversely, when the animal explores novel objects, the subiculum would encode recognition signals while probably coupling its activity with perirhinal and postrhinal networks and interrupting the flow from CA1, leading to low CA1–DS coherence. This model makes the testable prediction that the DS would display high coherence with perirhinal and postrhinal networks during object exploration and low coherence during spatial orientation.

An alternative proposal is that the subiculum might function by *integrating* the disparate spatial and recognition signals. In fact, Jacobs and Schenk (2003) have explicitly considered an integrator function for the subiculum within the context of their parallel map theory. In their view, the subiculum mediates the reference memory of an integrated map of space, which is built from two parallel systems, termed the bearing map and the sketch map. The authors speculate that the properties of subicular place units support such integrator function. Our results on DS place units are indeed compatible with this proposal of subicular function, insofar as it refers to spatial processing. Our data, however, has not uncovered an obvious mechanism that integrates the spatial and recognition codes within the subiculum. Such an integrator role could be implemented, for instance, by a set of spatially-selective cells that are also modulated by novelty. Given that we have not sampled the DS extensively, the presence of such subicular neurons that combine both codes remains a possibility.

We think our study is relevant for the conceptual discussion on the neural substrate of recognition memory. Our data are clearly not compatible with a strict version of the dual-process model that posits a complete segregation between the parahippocampal region encoding familiarity and the hippocampus encoding recollection (Eichenbaum et al., 1996; Yonelinas, 1999; Brown and Aggleton, 2001). According to this strict view, the subiculum (as the final station in the hippocampus) should not encode any novelty/familiarity signals, which our data obviously shows not to be the case. Instead, our results favor a softer version of the dual-process model, or even a single-process model (Shimamura, 2010; Voss and Paller, 2010), in which the subiculum contributes actively to the recognition of objects.

In conclusion, we used the NOR task that produced robust, spontaneous object exploration in mice to study whether DS participated in novelty recognition. We found that DS-theta power was modulated by object novelty and that DS showed high theta-gamma coherence with CA1 during spatial exploration, which decreased significantly when mice explored objects. We also found that DS pyramidal neurons were spatially selective and that a subset of DS cells responded specifically to novel object exploration. Our findings highlight a role for DS in encoding spatial and recognition-related signals, which match the recognized connectivity between the DS, the CA1 field, and the perirhinal cortex.

### Conflict of interest statement

The authors declare that the research was conducted in the absence of any commercial or financial relationships that could be construed as a potential conflict of interest.
